# Unexpected adrenal pheochromocytoma associated with a generalized tonic-clonic seizure in a prepubertal boy: A case report

**DOI:** 10.1097/MD.0000000000024303

**Published:** 2021-01-29

**Authors:** Zlatan Zvizdic, Mirsad Selimovic, Amira Mesic, Dusko Anic, Verica Misanovic, Faruk Skenderi, Semir Vranic

**Affiliations:** a Clinic of Pediatric Surgery; b Clinic of Urology; c Department of Anesthesiology and Reanimation; d Pediatric Clinic; e Department of Pathology, University Clinical Center Sarajevo, Sarajevo, Bosnia and Herzegovina; f College of Medicine, QU Health, Qatar University, Doha, Qatar.; g Biomedical and Pharmaceutical Research Unit, QU Health, Qatar University, Doha, Qatar.

**Keywords:** adrenal gland, children, Pheochromocytoma, seizure, treatment

## Abstract

**Rationale:**

Pheochromocytoma (PHEO) is a rare neuroendocrine tumor arising from chromaffin cells of the adrenal medulla. Most pediatric PHEOs are functional tumors, and clinical manifestations are related to catecholamine hypersecretion and/or tumor mass effects.

**Patient concerns:**

We report here a case of a 10-year-old boy with a highly functional adrenal PHEO detected after the evaluation of a generalized tonic-clonic seizure in the patient. His vital signs at admission were: blood pressure up to 220/135 mm Hg; pulse, 112 beats/min; temperature, 37.4°C; respiratory rate, 22 breaths/min.

**Diagnosis:**

A 24-hour urine collection for catecholamines test showed a marked increase in Vanillylmandelic acid levels (338.9 μmol/L). An abdominal magnetic resonance imaging revealed a well-defined left adrenal gland mass measuring ∼5 cm in its largest dimension.

**Interventions:**

The mass was surgically removed, and histopathological examination revealed PHEO with low malignant potential (Adrenal Gland Scaled Score/PASS/ < 4).

**Outcomes:**

The patient was discharged on the 10th postoperative day in good condition. At 24-month follow-up, the patient was doing well without complications such as tumor recurrence, elevated blood pressure, and seizure.

**Lessons:**

PHEO should be considered in the differential diagnosis of children with seizures presenting in the emergency department. A multidisciplinary approach to the evaluation and treatment of PHEO is also crucial for a successful outcome.

## 1 Introduction

Pheochromocytomas (PHEO) and extraadrenal paragangliomas are rare neuroendocrine tumors arising from chromaffin cells of the adrenal medulla or the sympathetic ganglia anywhere in the body. Approximately 10% to 20% of these tumors are diagnosed during childhood at an average age of 11 years with a slight male predominance, particularly under the age of 10.^[1]^

PHEOs are characterized by excessive amounts of catecholamines responsible for hypertensive surges, headache, and excessive sweating.^[2]^ The most common sign is hypertension, found in approximately 90% of patients with PHEO.^[3]^ Clinical characteristics of hypertension in PHEO patients vary and may show either a sustained or a paroxysmal pattern.^[4]^ More rarely, some patients present with hypotension, especially severe orthostatic with syncope.^[5,6]^ Exceptionally, a presenting symptom of PHEO may be seizures, as reported in a few cases in the literature.^[7,8]^

Here, we report a rare initial presentation of an adrenal PHEO in a previously healthy 10-year-old in whom a generalized tonic-clonic seizure is believed to be caused by the presence of an adrenal PHEO.

## 2 Case report

A 10-year-old boy was brought to our pediatric emergency department following an episode of a generalized tonic-clonic seizure. An emergency physician administered diazepam rectally. On initial physical examination, the patient was confused. The hetero-anamnestic data indicated that he had complained of the heat and sweating for several days before admission, mostly during the night. Nausea and vomiting occurred the morning before admission.

On the day of admission, his vital signs were as follows: Blood pressure, 145/85 mm Hg to 220/135 mm Hg; pulse, 112 beats/min; temperature, 37.4°C; respiratory rate, 22 breaths/min. Serum levels of electrolytes were all within normal limits. The abdomen was soft, painless to palpation, peristalsis was audible, peritoneal symptoms were absent. Cerebrospinal fluid was normal. An urgent computer tomography (CT) scan showed no brain lesions that could explain the decreased level of consciousness. An electroencephalogram was also normal. A 24-hour urine collection for catecholamines test showed a marked increase in Vanillylmandelic acid levels (338.9 μmol/L; normal values 8.1–37.8 μmol/L).

**Figure 1:**
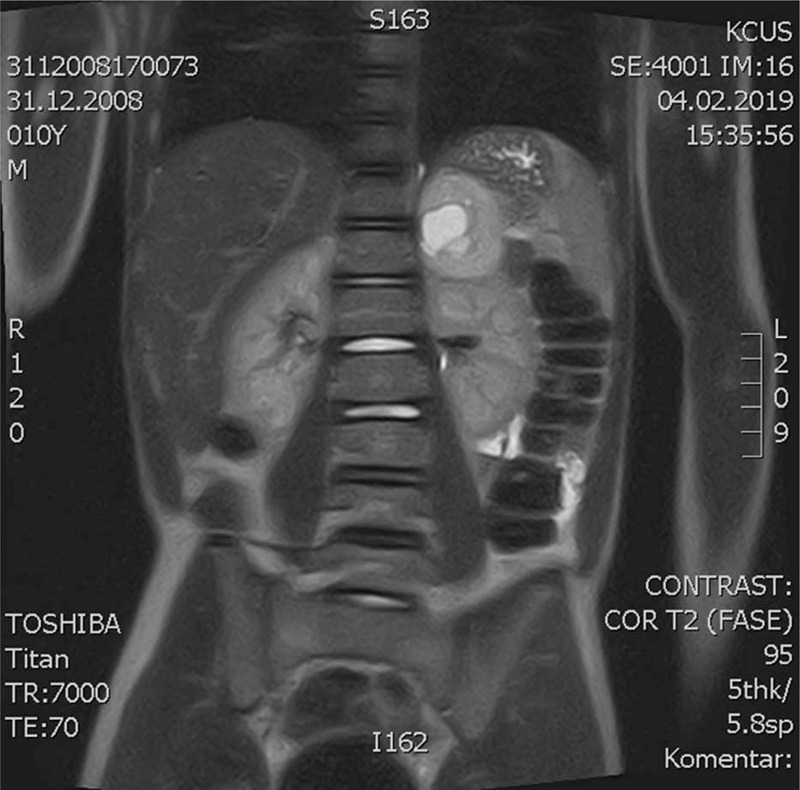
An abdominal magnetic resonance imaging revealed the left adrenal mass measuring 47.5 × 46.4 × 42 mm.

**Figure 2:**
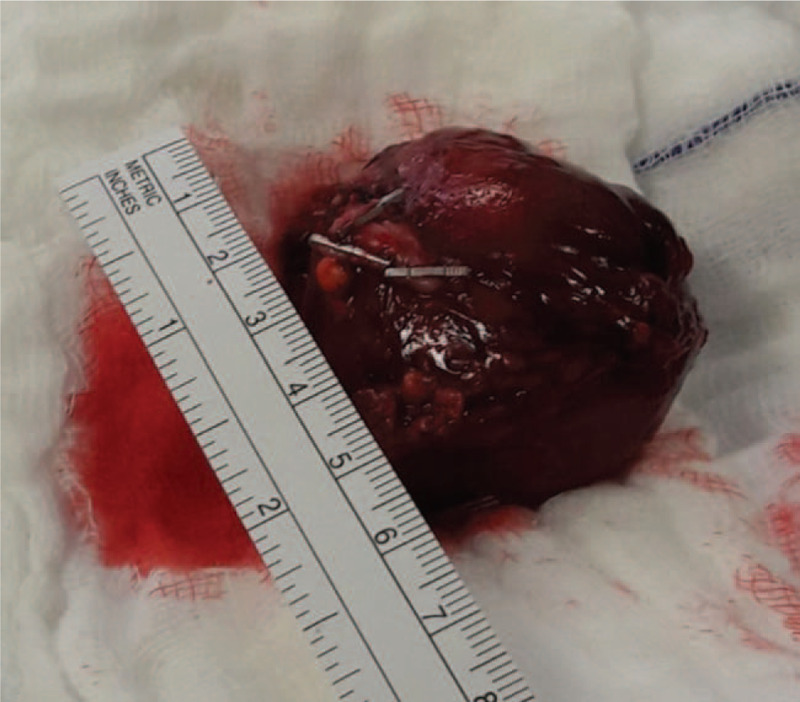
Gross intraoperative assessment of the resected tumor revealed a well-circumscribed solid mass with red to the brown hemorrhagic cut surface.

**Figure 3:**
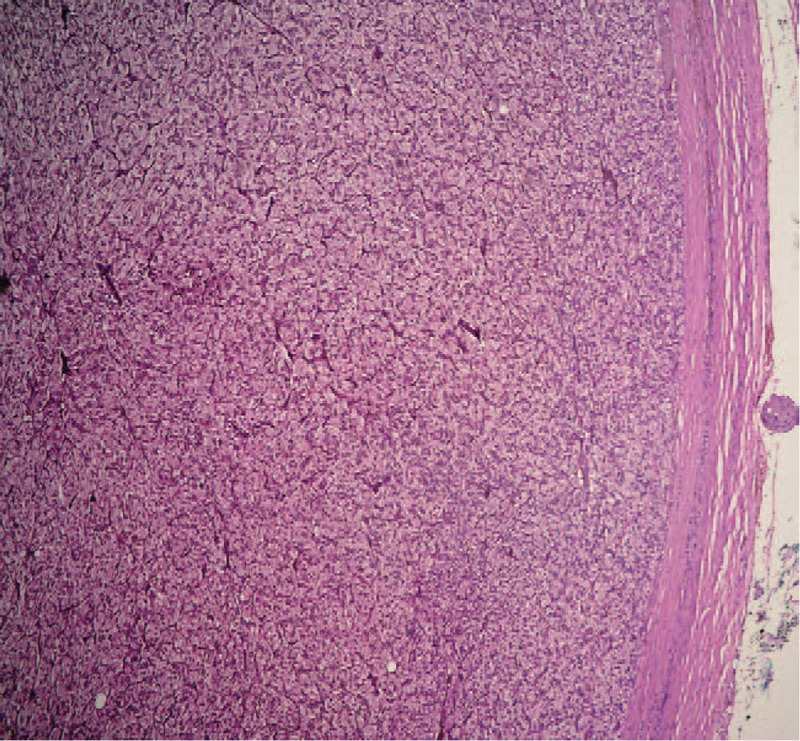
The histopathological assessment revealed a well-circumscribed mass composed of large, polygonal cells with abundant delicate, granular cytoplasm with a round to oval nuclei with prominent nucleoli (hematoxylin and eosin stain, ×10 magnification). The tumor cells were arranged in a solid and trabecular pattern. Adrenal Gland Scaled Score (PASS) was used to assess the neoplasm's malignant potential, revealing PASS <4 indicating a low malignant potential of the tumor.^[9]^

Abdominal ultrasound imaging revealed a heterogeneous solid mass measuring 48 × 40 mm in the left adrenal area, highly suspicious for an adrenal-originating tumor. An abdominal magnetic resonance imaging confirmed the presence of a well-defined left adrenal gland mass measuring 47.5 × 46.4 × 42 mm in its largest dimensions (Fig. 1). After rendering the provisional diagnosis of PHEO, in order to prevent hypertensive episodes and associated complications, we administered phenoxybenzamine as a nonspecific, alpha-blocking agent preoperatively to permit a vascular volume repletion and to block alpha-receptors and the expansion of intravascular volume before the surgery. After medical stabilization, a complete surgical excision of the adrenal mass was done through an open posterior approach without complications (Fig. 2). The histopathological examination of the surgical specimen was consistent with the diagnosis of an adrenal gland PHEO (Fig. 3).^[9]^ Adrenal Gland Scaled Score (PASS) was used to assess for malignant potential of the neoplasm revealing a PASS <4 and consequently indicating a low malignant potential of the tumor. Postoperatively, the patient developed a 2-day hypotension (average blood pressure of 80/50 mm Hg) with a heart rate of 100 bpm, which were effectively treated with noradrenaline drips. After that, the postoperative course was uneventful; the blood pressure was stabilized (on average 90/60 mmHg). The patient was discharged on the 10th postoperative day in a good condition. At 24-month follow-up, the patient was doing well without complications such as tumor recurrence, elevated blood pressure, and seizure.

## 3 Discussion

The symptoms and signs of PHEO are extremely variable, ranging from asymptomatic to cardiac arrest.^[4]^ Although the classic triad of symptoms in episodic headache, sweating, and palpitations is presented as typical, it occurs in only 24% of cases.^[4]^ Therefore, unusual presentations of PHEO seem to be more prevalent than previously thought. Various other symptoms that may manifest as episodic or continuous include severe anxiety, tremulousness, pain (chest, abdomen, lumbar, and groin), nausea, vomiting, weakness, fatigue, weight loss, warmth or heat intolerance, dyspnea, paresthesia, pallor of the face and upper body, and visual impairment.^[10]^ Rarely, PHEO may present with generalized tonic-clonic seizure and syncope due to orthostatic hypotension. Central nervous system manifestations are relatively rare in PHEO. Data from the literature suggest that the occurrence of seizures may be related to the high catecholamines, to hypertensive encephalopathy or brain tumors.^[11]^ Our patient had normal brain CT, and the seizures stopped with anticonvulsive and not with antihypertensive treatment. The presumed mechanism of seizure induction in our patient is possible cerebrovascular vasospasm due to markedly elevated catecholamines with consequent cerebral ischemia that provoked epileptic focus. Leiba et al^[7]^ provided a similar argument about the reason for seizures in patients with PHEO. Fitzgerald also suggests that norepinephrine, although known for its anticonvulsant properties, may also have proconvulsant properties under some conditions, both in animal epilepsy models and in humans.^[12]^ In addition, Pinheiro et al^[13]^ reported convulsive seizures simulating a PHEO resulting from an epileptogenic focus involving certain structures belonging to the autonomic nervous system and known as autonomic epilepsy. Finally, the association of carotid body paraganglioma and extraadrenal paraganglioma with the disappearance of epilepsy in the pediatric population has been reported.^[14,15]^ Rare cases of the seizures as the presenting symptoms of PHEO have been described in adults.^[7]^ To the best of our knowledge, this is the first case in the literature describing the seizures as a presenting symptom of an adrenal PHEO in a pediatric patient. Our comprehensive literature search revealed no similar cases reported in the pediatric population.

From all the above, it is clear that the diagnosis of PHEO is sometimes significantly aggravated in cases of its atypical and unusual clinical presentation, especially in the pediatric population, as confirmed in our case.

In conclusion, PHEO should be considered in the differential diagnosis of children presenting with seizures at the emergency departments. All unexplained conditions presenting with seizures and hypertension should have a high index of suspicion for the presence of PHEO in both adults and children, requiring a multidisciplinary approach to evaluation and treatment.

## Author contributions

**Conceptualization:** Zlatan Zvizdic, Semir Vranic.

**Data curation:** Zlatan Zvizdic, Mirsad Selimovic, Semir Vranic, AM.

**Formal analysis:** Zlatan Zvizdic, Mirsad Selimovic, Amira Mesic, Dusko Anic, Verica Misanovic, Faruk Skenderi, Semir Vranic.

**Funding acquisition:** Semir Vranic.

**Investigation:** Zlatan Zvizdic, Mirsad Selimovic, Amira Mesic, Dusko Anic, Verica Misanovic.

**Writing – original draft:** Zlatan Zvizdic, Semir Vranic.

**Writing – review & editing:** Semir Vranic, Zlatan Zvizdic.

## Abbreviations

Abbreviations: CT = computed tomography, PASS = Adrenal Gland Scaled Score, PHEO = pheochromocytoma.
